# Cap‐independent translation of GPLD1 enhances markers of brain health in long‐lived mutant and drug‐treated mice

**DOI:** 10.1111/acel.13685

**Published:** 2022-08-05

**Authors:** Xinna Li, Xiaofang Shi, Madaline McPherson, Mary Hager, Gonzalo G. Garcia, Richard A. Miller

**Affiliations:** ^1^ Department of Pathology, School of Medicine University of Michigan Ann Arbor Michigan USA; ^2^ College of Literature, Sciences, & the Arts University of Michigan Ann Arbor Michigan USA; ^3^ University of Michigan Geriatrics Center Ann Arbor Michigan USA

**Keywords:** aging, cap‐independent translation, glycosylphosphatidylinositol specific phospholipase D1, growth hormone

## Abstract

Glycosylphosphatidylinositol‐specific phospholipase D1 (GPLD1) hydrolyzes inositol phosphate linkages in proteins anchored to the cell membrane. Mice overexpressing GPLD1 show enhanced neurogenesis and cognition. Snell dwarf (DW) and growth hormone receptor knockout (GKO) mice show delays in age‐dependent cognitive decline. We hypothesized that augmented GPLD1 might contribute to retained cognitive function in these mice. We report that DW and GKO show higher GPLD1 levels in the liver and plasma. These mice also have elevated levels of hippocampal brain‐derived neurotrophic factor (BDNF) and of doublecortin (DCX), suggesting a mechanism for maintenance of cognitive function at older ages. GPLD1 was not increased in the hippocampus of DW or GKO mice, suggesting that plasma GPLD1 increases elevated these brain proteins. Alteration of the liver and plasma GPLD1 was unaltered in mice with liver‐specific GHR deletion, suggesting that the GH effect was not intrinsic to the liver. GPLD1 was also induced by caloric restriction and by each of four drugs that extend lifespan. The proteome of DW and GKO mice is molded by selective translation of mRNAs, involving cap‐independent translation (CIT) of mRNAs marked by N^6^ methyladenosine. Because GPLD1 protein increases were independent of the mRNA level, we tested the idea that GPLD1 might be regulated by CIT. 4EGI‐1, which enhances CIT, increased GPLD1 protein without changes in GPLD1 mRNA in cultured fibroblasts and mice. Furthermore, transgenic overexpression of YTHDF1, which promotes CIT by reading m6A signals, also led to increased GPLD1 protein, showing that elevation of GPLD1 reflects selective mRNA translation.

## Graphical abstract

Two kinds of long‐lived mice, Snell dwarf and GHRKO, have higher levels of GPLD1 in liver and plasma, as well as higher levels of two GPLD1‐stimulated brain proteins, BDNF and DCX. Increased GPLD1 reflects selective cap‐independent mRNA translation rather than transcriptional changes. Last, GPLD1 can be increased, in cultured cells and in mice, by a transgene and by a drug that turns on cap‐independent translation.

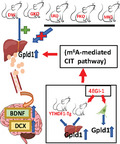

**Abbreviations**
BDNF: brain‐derived neurotrophic factor
CIT: cap‐independent translation
DCX: double cortin
DW: Snell dwarf
GHR: growth hormone receptor
GKO: growth hormone receptor knockout
GPLD1: glycosylphosphatidylinositol‐specific phospholipase D1
YTHDF1: YTH N6‐methyladenosine RNA binding protein 1

## 1 INTRODUCTION

The first published evidence that mutation in a specific gene could lead to a dramatic extension of lifespan in a mammal was the observation (Brown‐Borg et al., 1996) that Ames dwarf mice, lacking pituitary production of growth hormone (GH), thyroid stimulating hormone (TSH), and prolactin, lived >30% longer than littermate controls. Similar data for the Snell dwarf pituitary mutant (Flurkey et al., 2001) and GH receptor knock‐out (GHRKO) mice (Zhou et al., 1997) suggested that healthy longevity was often increased in mice with deficits in GH production or response, which also had lower levels of insulin‐like growth factor 1 (IGF‐1), produced by liver in response to GH signals. Additional lifespan experiments in Ghrhr‐deficient (Flurkey et al., 2001; Sun et al., 2013) and Ghrh‐deficient mice (Bartke et al., 2021; Icyuz et al., 2020) were consistent with this idea. Increased longevity in mice with mutations in Pappa (Bale & Conover, 2005), an enzyme that can increase IGF‐1 action by cleavage of IGF‐1 binding proteins, implied that modulation of local IGF‐1 levels could be involved in the anti‐aging effects of GH and GHR mutants. Studies of flies and worms with defects in production of, or response to, insulin‐like hormones suggested that the linkage of insulin and lifespan had deep evolutionary roots (Clancy et al., 2001; Kimura et al., 1997; Lin et al., 1997). There is now ample evidence that the longevity seen in these mutant mice is usually accompanied by preservation of many age‐sensitive functional capacities and avoidance of age‐dependent forms of lethal and non‐lethal pathology (Aberg et al., 2000; Dominick et al., 2017; Flurkey et al., 2001; Ikeno et al., 2003, 2009).

In rodents and in humans, aging leads to decline in memory and other aspects of cognitive function (Gemma & Bickford, 2007; Harada et al., 2013; Leal & Yassa, 2015), and there is evidence for Ames and GHRKO mice that the increase in lifespan is accompanied by a slower rate of decline in cognitive function (Kinney, Meliska et al., 2001; Sun, Al‐Regaiey et al., 2005; Sun, Evans et al., 2005). Little is known about the mechanisms by which GH deficiency or GH resistance could delay mental aging together with delay of other forms of late‐life illness and death.

The abundance of GHR in the CNS suggests a connection between GH or IGF‐1 signals and CNS physiology (Ashpole et al., 2015). GH receptors can be found in the thalamus, putamen, and hippocampus, and studies have identified the effects of GH in the CNS and peripheral nervous system (PNS). The GH and IGF‐1 axis, as well as IGF‐2, affect early brain development, maturation, and function. An early study of Ames dwarf mice (Sun, Al‐Regaiey et al., 2005) showed that the hippocampus of these mice had elevation of both GH and IGF‐1 compared to littermate controls, with corresponding elevation of the PI3K‐Akt‐CREB cascade, and the same group also found (Sun, Evans et al., 2005) increased neurogenesis in the dentate gyrus of Ames dwarf adults compared to controls. This local production of GH and IGF‐1 may lead to higher levels of neural cell generation in these mice, possibly reflecting lower levels of GH and IGF‐1 in the peripheral circulation. Mechanisms that might contribute to alterations in CNS neural cell survival and turnover in long‐lived mice have not yet been delineated.

Glycosylphosphatidylinositol‐specific phospholipase D (GPLD1) is a 110‐kDa amphiphilic N‐glycosylated protein in mammalian plasma that interacts with high‐density lipoproteins (HDL). GPLD1 has not only been found to be derived primarily from the liver but may also be produced in the brain, kidney, muscle, immunocytes, and inflammatory cells (Qin et al., 2016). GPI phospholipases are able to cleave many glycosylphosphatidylinositol (GPI)‐anchored proteins. A recent study linked blood levels of GPLD1 to an improvement of brain activity in older mice (Horowitz et al., 2020). This study showed that exercise led to increased levels of GPLD1 in mouse blood, a correlation also seen in active elderly humans. The exercised mice in this experiment showed improved performance on both a radial arm water maze test and in tests of contextual fear conditioning (Horowitz et al., 2020). In vivo transfection was then used to increase Gpld1 in the liver of aged mice, leading to increased plasma Gpld1, an increase in adult neurogenesis and higher expression of BDNF in the hippocampus of aged mice (Horowitz et al., 2020).

Brain‐derived neurotrophic factor (BDNF) is a member of the neurotrophin family of proteins and regulates synaptic plasticity, synaptogenesis, neuronal cell survival, and neurogenesis (Greenberg et al., 2009; Park & Poo, 2013). BDNF also affects other aspects of brain function such as learning, memory, and cognition (Kuipers & Bramham, 2006; Loprinzi & Frith, 2019). Aging leads to lower BDNF production in the hippocampus (Romo‐Araiza et al., 2018) and also reduces the amount of BDNF circulating in plasma (Bednarski et al., 1998; MacPherson, 2017). Doublecortin (DCX), found in developing and adult mammal neuroblasts, is a microtubule‐associated protein (Couillard‐Despres et al., 2005; Francis et al., 1999; Gleeson et al., 1999). In the adult brain, DCX expression is used as an indicator of neural progenitor cells (NPCs), which generate new neurons in the dentate gyrus (Balthazart & Ball, 2014).

To explore routes by which mice with low GH or GH response might preserve cognitive function and neurogenesis in adult life, we measured GPLD1, BNDF, and DCX in the liver, brain, and plasma of Snell dwarf and GHRKO mice. To distinguish between direct and indirect GH effects, we also evaluated mice with disruption of GHR in the liver (LKO), muscle (MKO), or fat (FKO). Derivation and physiological characteristics of these five mouse models have been described previously (List et al., 2013, 2014, 2015; Zhou et al., 1997).

We also evaluated whether changes in GPLD1 were regulated by alterations in mRNA levels or by changes in translation of mRNA to GPLD1 protein. mRNA translation in eukaryotes typically involves binding of initiation factors to the 5‐prime cap structure (Decroly et al., 2011), but a subset of mRNAs can be translated by a cap‐independent mechanism (CIT), which is less well‐studied. CIT plays an important role in cell differentiation, influences cellular response to nontypical conditions, and modulates metabolic processes, stress resistance, and development (Dennis et al., 2013; Lacerda et al., 2017; Shatsky et al., 2018). Recognition and translation of mRNAs for CIT depends, in part, on a protein, YTHDF1, that binds to N^6^‐methyladenosine (m6A) residues in the 5′‐untranslated region of CIT‐susceptible RNAs. Garcia et al. have shown increased translation of specific mRNAs *via* CIT pathways both in long‐lived mutant mice (Ozkurede et al., 2019) and in mice in which drugs were used to increase lifespan (Shen et al., 2021), suggesting that some proteins may increase in long‐lived mice by differential, cap‐independent, mRNA translation.

## 2 RESULTS

### 2.1 Long‐lived mutant mice have higher levels of hepatic GPLD1

**FIGURE 1:**
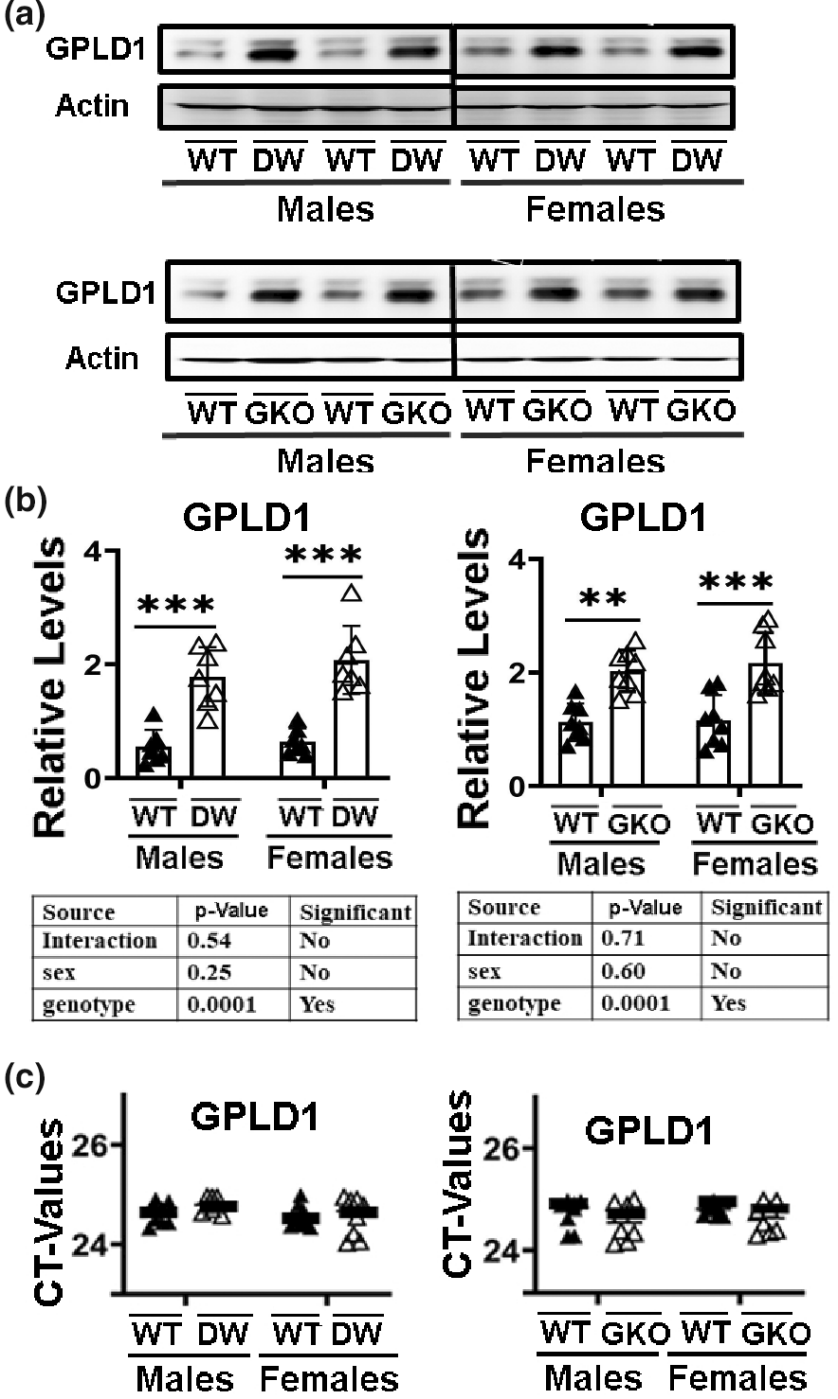
Expression of GPLD1 in liver tissue of Snell dwarf mice (DW) and growth hormone receptor (GHR) knockout mice (GKO). (a) Cell lysate was prepared from liver tissues of 24‐week‐old WT and GKO mice. Protein levels of liver GPLD1 were evaluated by Western blotting. Representative gel images are shown. (b) Relative protein expression was normalized to *β‐actin* levels. Values are mean ± SEM (*n* = 8). **p < 0.01 and ***p < 0.001 versus WT. (c) Total RNAs were isolated from liver tissues of 24‐week‐old wild type littermate control mice (DWWT or GKOWT) and long‐lived mice (DW or GKO). mRNA levels of *GPLD1* were measured by qRT‐PCR. No significant differences were found in the mean CT value (horizontal bar) of the corresponding mRNA levels between control and long‐lived mice (*n* = 8). Each symbol represents a different mouse.

We evaluated *GPLD1* mRNA and protein levels in liver of young adult (24‐week‐old) mice of two kinds of long‐lived mutants (DW and GKO) and their littermates as controls (DWWT or GKOWT). We evaluated eight males and eight females of each mutant genotype and an equal number of control mice. GPLD1 protein levels were significantly elevated in the liver of each variety of long‐lived mice (DW or GKO) compared to littermate control mice (*p* < 0.001) (Figure 1a,b). Two‐factor ANOVA, taking sex and genotype as predictors, found no sex effect on GPLD1 expression, and no [sex × genotype] interaction; the effect of DW and GKO is equivalent in males and females. In contrast, we found no significant differences between mutants and controls in mRNA levels (Figure 1c). The effect on protein levels in the absence of changes in mRNA suggests that levels of GPLD1 protein may be modulated by a posttranscriptional mechanism.

### 2.2 GPLD1 is increased in the plasma of long‐lived mice

**FIGURE 2:**
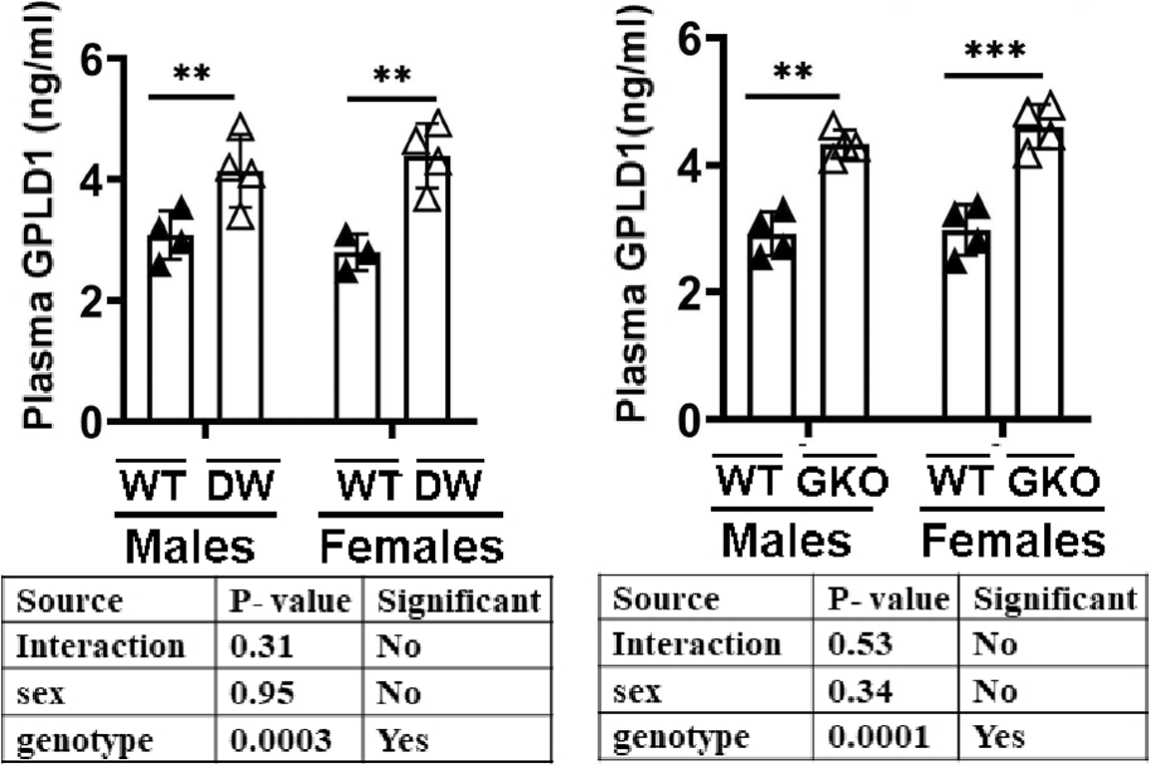
Plasma GPLD1 levels of WT (DWWT or GKOWT) and mutant mice (DW or GKO). GPLD1 content was measured by ELISA assay on plasma samples of 24‐week‐old wild type littermate control mice (DWWT or GKOWT) and long‐lived mice (DW or GKO). Data are shown as mean ± SEM for each group (*n* = 4). **p < 0.01 and ***p < 0.001 versus WT.

GPLD1 produced in the liver is secreted into the blood (LeBoeuf et al., 1998). Consistent with our data on liver GPLD1, we found elevated levels of GPLD1 in plasma of DW and GKO mice, with no indication of sex‐specific effects (Figure 2). We also measured GPLD1 in plasma of liver‐specific GHR knock‐out (LKO) mice, which are not long‐lived (List et al., 2014), to see if the effect of the global GKO represented a direct effect of GH signals on the liver. Interestingly, GPLD1 was not higher in plasma of LKO mice, suggesting that the DW and GKO effects probably do not involve GH signals to liver cells per se (Figure S1a).

We have previously found alterations in fat depot function in GKO mice, including alterations in thermogenesis, ratio of inflammatory to anti‐inflammatory macrophages, and cell size, and shown that these reflect indirect effects of GH on muscle cells, rather than effects of GH on fat cells or changes in liver‐produced IGF‐1 (Li et al., 2020). We therefore evaluated plasma GPLD1 in mice with muscle‐specific knock‐out of GHR (Figure S1c) and found no effect on GPLD1 levels in the MKO mice. Mice with fat‐specific disruption of GHR also had levels of plasma GPLD1 similar to their littermate controls (Figure S1b), Consistent with the plasma results, there were no changes in GPLD1 mRNA or protein in the liver of LKO, MKO, or FKO mice (Figure S2). Thus, the effects of global GKO appear not to reflect GH effects directly on the liver, muscle, or fat.

### 2.3 BDNF is increased in the hippocampus of DW and GKO mice

**FIGURE 3:**
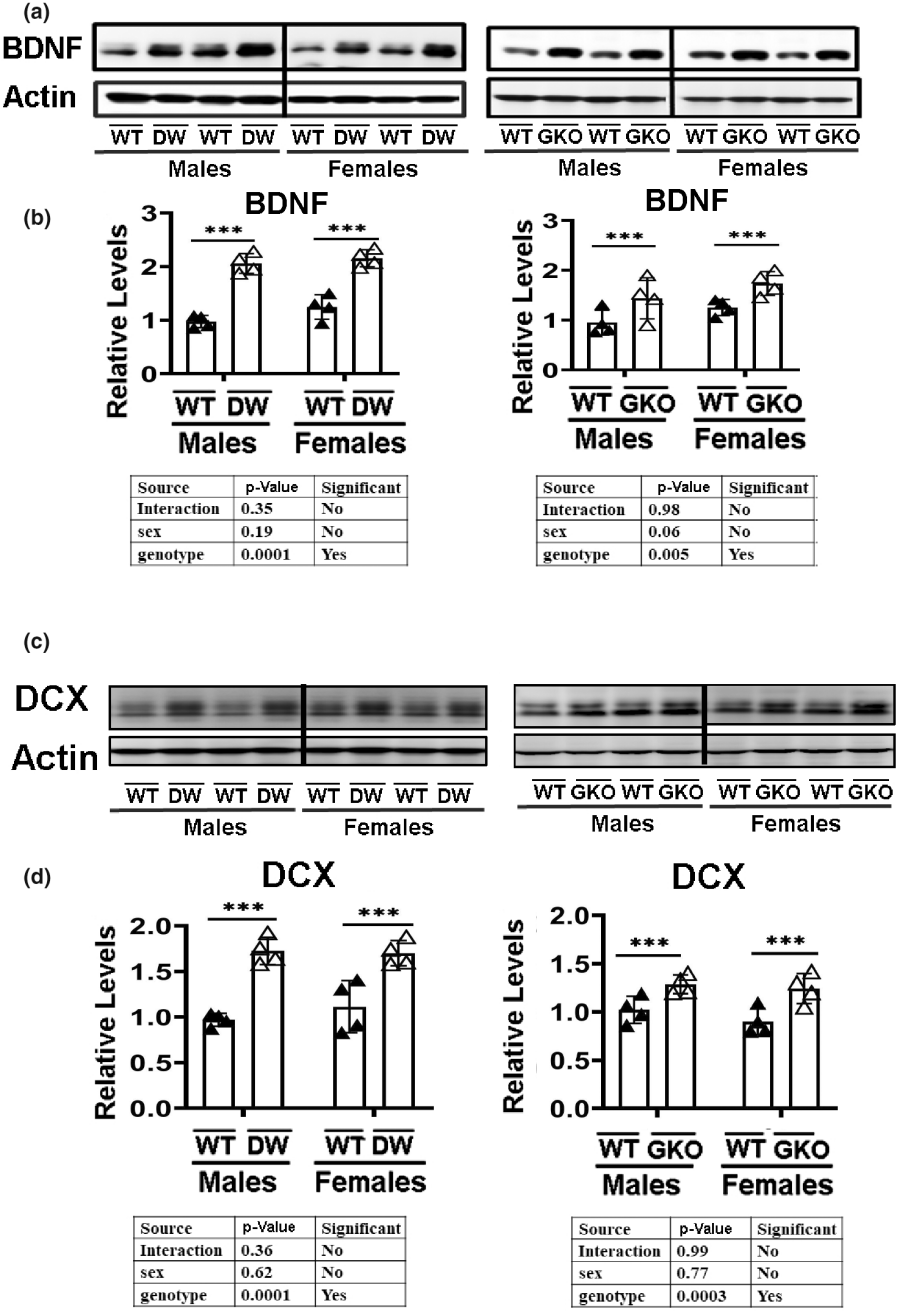
Expression of BDNF and doublecortin (DCX) in hippocampus of Snell dwarf mice (DW) and growth hormone receptor knockout mice (GKO). (a) Cell lysate was prepared from the hippocampus of 24‐week‐old wild type littermate control mice (DWWT or GKOWT) and long‐lived mice (DW or GKO). Protein levels of BDNF were then measured by Western blotting. Representative gel images are shown. (b) Relative protein expression was normalized to β‐actin levels. Values are mean ± SEM (*n* = 4). ***p < 0.001 versus WT. (c) Cell lysate was prepared from hippocampus tissues of 24‐week‐old wild type littermate control mice (DWWT or GKOWT) and long‐lived mice (DW or GKO). Protein levels of DCX were then measured by Western blotting. Representative gel images are shown. (d) Relative protein expression was normalized to β‐actin levels. Values are mean ± SEM (*n* = 4). ***p < 0.001 versus WT.

The brain‐derived neurotrophic factor (BDNF) belongs to a family of neurotrophins and is critical to neuronal changes involved in learning and memory (Miranda et al., 2019). Both Ames dwarf (highly similar to Snell dwarf) and GKO mice have been shown to retain good cognitive function at ages where controls show cognitive declines (Ayanlaja et al., 2017; Hascup et al., 2017). Liver‐derived GPLD1 has been shown to improve function in the hippocampus of aged mice (Horowitz et al., 2020) and thus might in principle contribute to retention of cognition in aging DW and GKO mice. We therefore examined the expression levels of BDNF in the hippocampus by Western blot and found elevated expression in both DW and GKO mice (Figure 3a,b), with no evidence for sex‐specific differences. The increase was modest, i.e., 50% to 100% elevation, but statistically significant at *p* < 0.005 for each mouse model. Although we have not yet measured GPLD1 or BDNF in aged mice of either genotype, these results hint that elevation of GPLD1 and BDNF, either in young mice or throughout the lifespan, could lead to preservation of hippocampal function and cognitive performance in mice of these slow‐aging genotypes.

### 2.4 Doublecortin is increased in the hippocampus of DW and GKO mice

Doublecortin is an essential factor in neurogenesis and is considered a marker of newly generated cells in the CNS (Ayanlaja et al., 2017). Immunoblotting data revealed higher levels of doublecortin (Dcx) in the hippocampus of both male and female GKO mice (*p* < 0.001) (Figure 3c,d). DW mice showed similar effects. In each case, the size of the effect – about 50% – is modest, but statistically significant at *p* < 0.0003, and is seen in mice of both sexes.

### 2.5 Tissue‐specific deletion of GHR in the liver, muscle, or fat does not alter hippocampal BDNF or DCX

Because plasma levels of LKO, MKO, and FKO mice did not show any changes in GPLD1, we expected that these three stocks would not show any changes in hippocampal BDNF or DCX. Results are shown in Figures S3 and S4. Consistent with the plasma GPLD1 results, neither BDNF nor DCX protein was changed in any of these tissue‐specific KO models.

### 2.6 There is no change in expression of GPLD1 in hippocampus of long‐lived mutant mice

**FIGURE 4:**
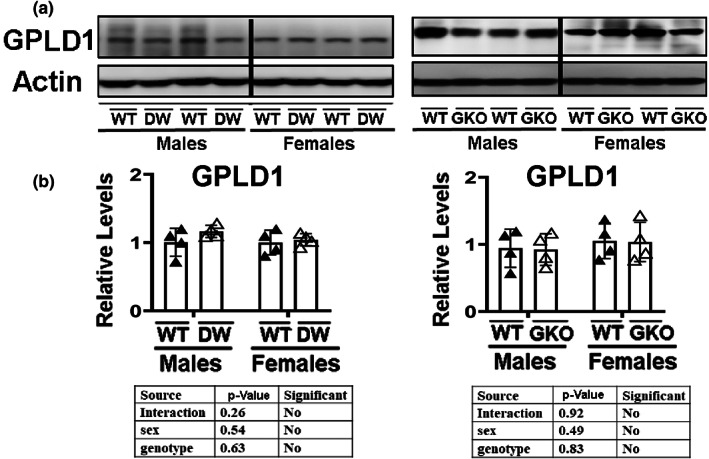
Expression of GPLD1 in hippocampus of Snell dwarf mice (DW) and growth hormone receptor knockout mice (GKO). (a) Cell lysate was prepared from the hippocampus of 24‐week‐old wild type littermate control mice (DWWT or GKOWT) and long‐lived mice (DW or GKO). Protein levels of GPLD1 were then measured by Western blotting. Representative gel images are shown. (b) Relative protein expression was normalized to β‐actin levels. Values are mean ± SEM (*n* = 4).

Although hippocampal expression of BDNF and DCX is thought to reflect effects of peripheral, i.e., plasma, GPLD1 (Horowitz et al., 2020), GPLD1 is produced within the brain as well (LeBoeuf et al., 1998). We therefore tested whether levels of GPLD1 might be higher in the hippocampus of DW or GKO mice. We observed no changes in hippocampal GPLD1 protein in either DW or GKO mice compared to their respective controls (Figure 4) nor did we see any alteration of hippocampal GPLD1 in LKO, MKO, or FKO mice (Figure S5).

### 2.7 Effects of 4EGI‐1 on levels of GPLD1 in cultured, skin‐derived mouse fibroblasts

**FIGURE 5:**
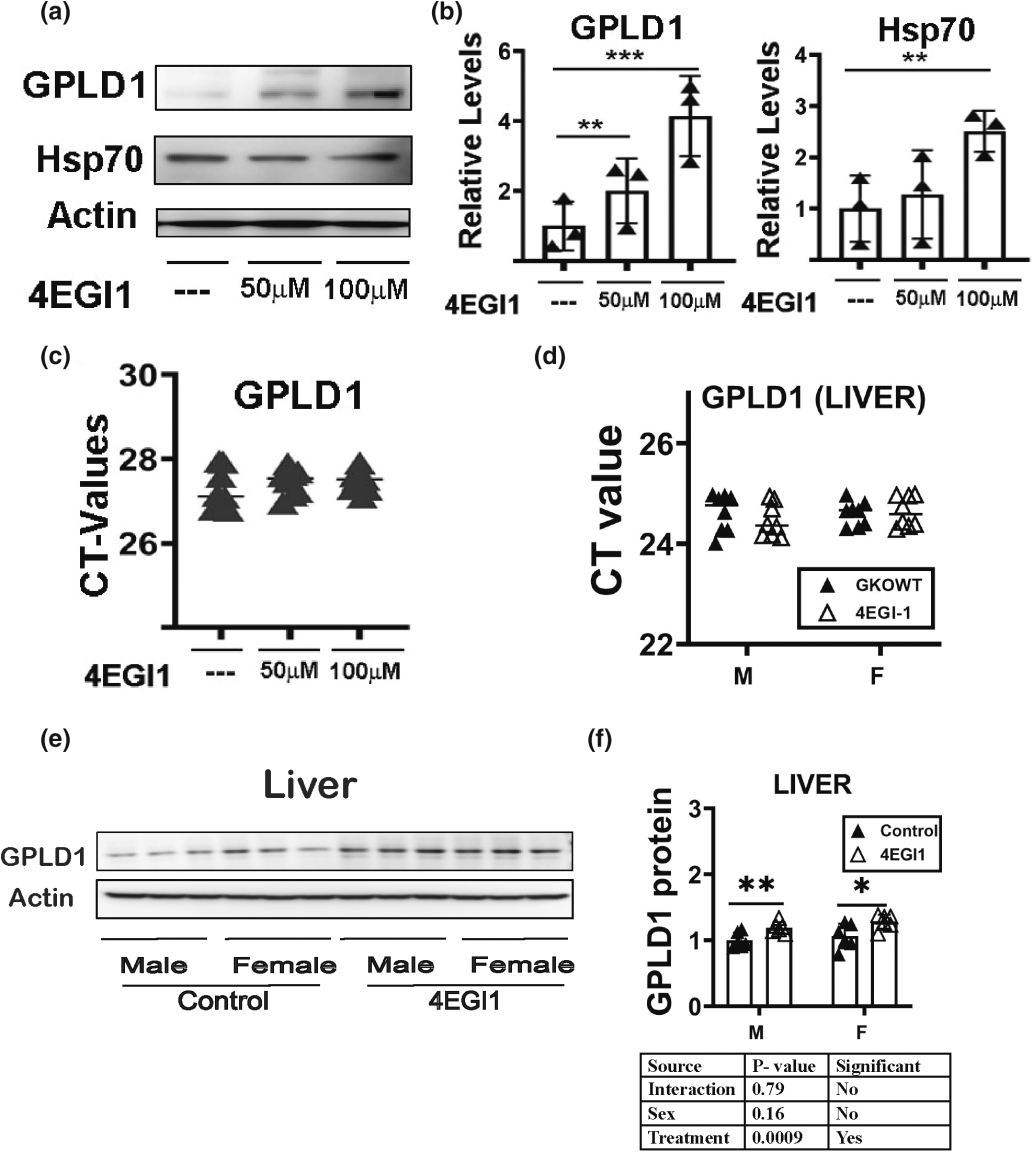
Effects of 4EGI‐1 on levels of GPLD1 in cultured, skin‐derived mouse fibroblasts. (a) Representative Western blot analysis of 4EGI‐1 effects in UM‐HET3 mouse fibroblast cells. DMSO is the vehicle control. Two replicates are shown for each of two doses of 4EGI‐1. (b) Bars represent the mean ± SEM from fibroblasts of three mice per group. Values are mean ± SEM (*n* = 3). **p < 0.01 versus DMSO, and ***p < 0.001 versus DMSO. (c) CT values of qRT‐PCR for GPLD1 performed using at least eight control and eight 4EGI‐1 fibroblast preparations per group. (d) Total RNAs were isolated from liver tissues of 24‐week‐old control and 4EGI‐1 treated mice. mRNA levels of *GPLD1* were measured by qRT‐PCR. (e) Cell lysate was prepared from liver tissues of 24‐week‐old control and 4EGI‐1 treated mice. Protein levels of liver GPLD1 were evaluated by Western blotting. Representative gel images are shown. (f) Relative protein expression was normalized to *β‐actin* levels. Values are mean ± SEM (*n* = 6). *p < 0.05 and **p < 0.01versus controls.

Translation of most mRNAs is dependent on a group of translation initiation factors that bind to the m7G cap structure at the 5′ end of the mRNA, a process termed cap‐dependent translation (Jackson et al., 2010). Several forms of cellular stress can inhibit cap‐dependent translation and trigger an alternative mechanism, termed cap‐independent translation (CIT), requiring recognition of N^6^‐methyladenosine markers in the RNA's 5′‐untranslated sequence (Spriggs et al., 2010). Only a subset of mRNAs can be translated *via* CIT. Previous studies in DW and GKO mice have shown that CIT is elevated in the liver of these mice, and multiple proteins whose mRNA is CIT‐susceptible are present in elevated amounts in DW and GKO mice (Ozkurede et al., 2019). The increase of GPLD1 protein in the absence of increased GPLD1 mRNA suggested that GPLD1 mRNA may be translated through CIT. We have previously shown elevation of several CIT‐target proteins in fibroblasts and in mice in response to 4EGI‐1 (Moerke et al., 2007; Ozkurede et al., 2019). We treated normal mouse fibroblasts with 4EGI‐1 for 24 h and found that GPLD1 protein increased in a dose‐dependent fashion (Figure 5a,b). Hsp70 is reported to be translated through cap‐independent translation and was used as a positive control for 4EGI‐1 effect (Meyer et al., 2015; Zhou et al., 2015; Zou et al., 2015). As expected, 4EGI‐1 did not alter mRNA encoding GPLD1 (Figure 5c). These data suggest that GPLD1 can indeed be increased by CIT, at least in cultured fibroblasts. We then evaluated GPLD1 levels in the liver of mice that had been given daily doses of 4EGI‐1 by gavage for 7 days. As expected, 4EGI‐1 did not increase liver mRNA levels for GPLD1 (Figure 5d). GPLD1 protein, however, was increased in the liver (Figure 5e,f) by 4EGI‐1; the effect, though small, was significant at *p* < 0.001, and was seen in both sexes. These results, like the data from 4EGI‐1 treated fibroblasts, indicate that increased CIT leads to increased GPLD1 protein without an effect on mRNA levels for this protein.

### 2.8 Higher GPLD1 levels in liver of YTHDF1 transgenic mice with elevated CIT

**FIGURE 6:**
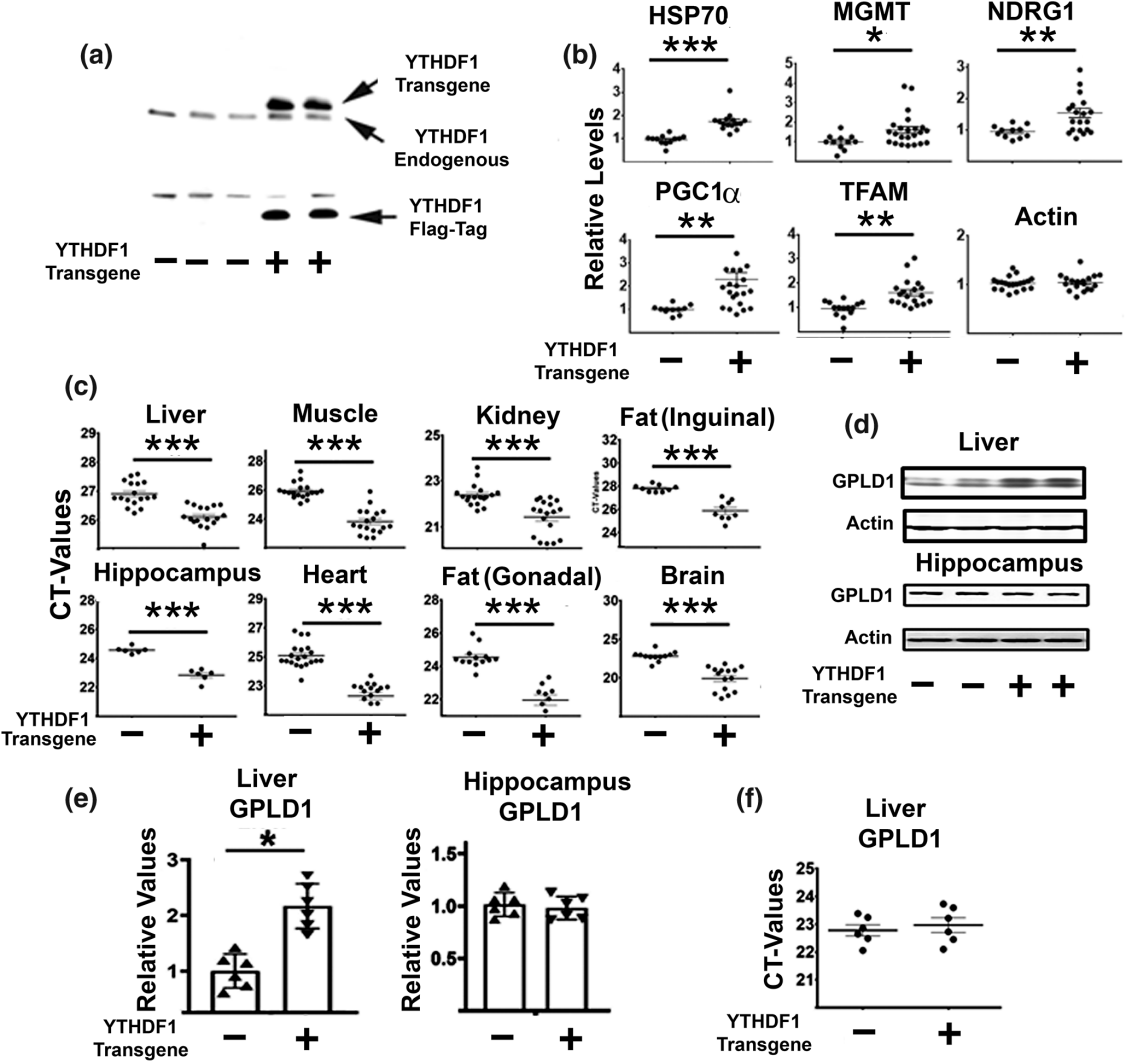
Liver from YTHDF1 transgenic mice shows upregulation of GPLD1 protein levels. (a) Representative Western blot in liver samples against YTHDF1 and Flag‐Tag from negative (endogenous YTHDF1) and positive YTHDF1‐Flag‐Tag (+) transgenic. (b) Mean ± SEM of level of CIT dependent proteins in liver samples from at least eight negative and eight positive YTHDF1 transgenic mice. All extracts were normalized to actin. *Represent statistical significance *p* < 0.05. (c) Levels of YTHDF1 mRNA in different tissues from at least eight negative and eight positive YTHDF1 transgenic mice. *Represent statistical significance *p* < 0.05. (d) Mean ± SEM of GPLD1 protein in liver samples normalized by actin from at least four negative and four positive YTHDF1 transgenic mice. (e) Relative protein expression normalized to β‐actin levels. Values are mean ± SEM (*n* = 6). **p < 0.01 versus WT. (f) Total RNAs were isolated from liver tissues of 24‐week‐old wild type littermate control mice (WT) and YTHDF1 transgenic mice. mRNA levels of *GPLD1* were measured by qRT‐PCR. No significant differences were found in the mean CT value (horizontal bar) of the corresponding mRNA levels between control and YTHDF1 transgene mice (*n* = 6). Each symbol represents a different mouse.

We have reported that YTHDF1 protein levels are increased in the liver, kidney, and muscle of DW and GKO mice (Ozkurede et al., 2019). YTHDF1 is a protein involved in enhancing cap‐independent translation (CIT) of selected mRNAs containing N^6^‐methyladenosine (m6A) sites in the 5′‐untranslated region (Meyer et al., 2015; Wang et al., 2014, 2015; Zhou et al., 2015). Because we found enhanced expression of GPLD1 protein levels in the liver of the DW and GHKO without changes in their mRNAs (Figure 1), we hypothesized that tissue upregulation of YTHDF1 in mice would lead to elevated GPLD1 protein *via* increased CIT. To test this hypothesis, we constructed a YTHDF1 overexpressing transgenic mouse as described in Section 4 (Figure S1d). The mice are fertile and transmit the transgene to offspring. We found that YTHDF1 is overexpressed in multiple tissues (Figure 6a,c) using Western blots or qRT‐PCR. These mice show no evident developmental or physiological abnormalities for at least the first 6 months of age. YTHDF1 overexpression does lead, in a fraction of the mice (about 25%), to a behavioral abnormality, i.e., a tendency to walk in a circle, a trait not associated with changes in brain structure (Garcia and Miller, unpublished data). We expected that these YTHDF1 transgenic mice would show upregulation of proteins dependent on CIT (Meyer et al., 2015; Wang et al., 2014, 2015; Zhou et al., 2015), including those we have already documented to be elevated in long‐lived mutant mice (Dominick et al., 2017; Ozkurede et al., 2019), a set which includes MGMT, TFAM, PGC1α, NDRG1, and Hsp70. Indeed, as predicted, liver tissues from the YTHDF1 overexpressing mice show higher levels of all five of these proteins (Figure 6b), consistent with upregulation of CIT. Liver of YTHDF1 transgenic mice also had higher levels of GPLD1 protein, compared to their littermate controls, without any change in GPLD1 mRNA, consistent with elevation of other CIT proteins in these mice (Figure 6e,f). YTHDF1 overexpression did not alter levels of GPLD1 in the hippocampus (Figure 6e), consistent with the lack of effects on GPLD1 in the brain of Snell and GKO mice. The basis for the tissue‐specific effects of YTHDF1 on GPLD1 translation is not known but presumably reflects tissue‐specific differences in the machinery of mRNA selection, compartmentalization, and translation, alterations in addition of m6A residues in specific mRNAs, or levels of proteins that interact with YTHDF1 to control translation of mRNA subsets. Elevation of GPLD1 translation in livers of the DW and GKO mice could reflect the higher levels of YTHDF1 in these mice, perhaps along with other factors that favor translation by CIT over translation of mRNAs by cap‐dependent pathways.

### 2.9 Sex‐specific elevation of GPLD1 by drugs that increase mouse lifespan, and by caloric restriction (CR)

**FIGURE 7:**
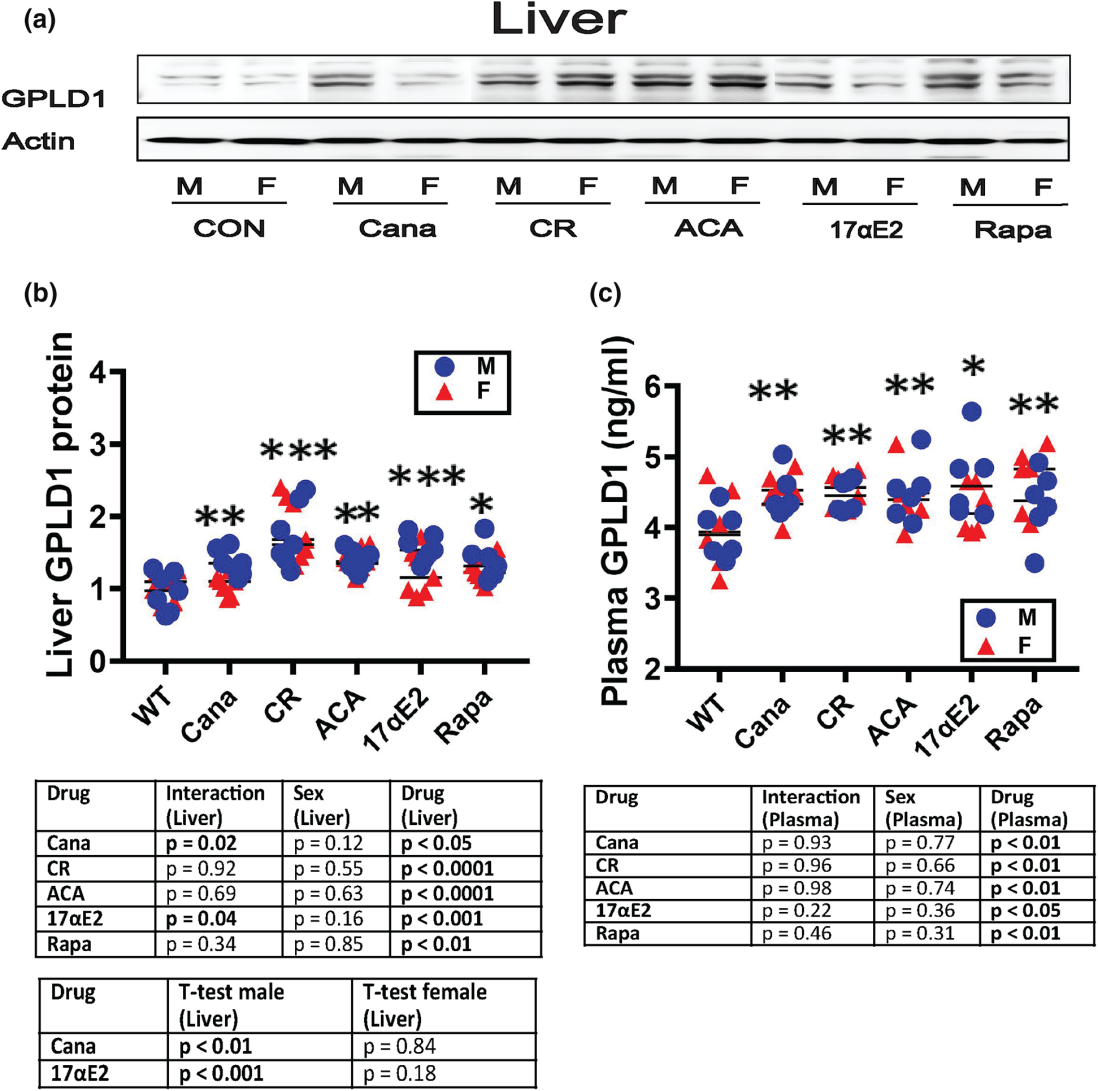
Effects of diet treatment (Cana, CR, ACA, 17aE2 and Rapa) on the expression of GPLD1 in liver and plasma. (a) Total protein were isolated from liver of 48‐week‐old wild type littermate control mice (Con) and diets treated mice (Cana, CR, ACA, 17aE2, and Rapa). Protein levels of liver GPLD1 were evaluated by Western blotting. Representative gel images are shown. (b) Relative GPLD1 protein expression was normalized to β‐actin levels. Values are mean ± SEM (*n* = 7). *p < 0.05 versus Con. **p < 0.01 versus Con and ***p < 0.001 versus Con. Statistics table of GPLD1 protein levels in liver. (c) GPLD1 content was measured by ELISA assay on plasma samples of 48‐week‐old wild type littermate control mice (Con) and diet‐treated mice (Cana, CR, ACA, 17aE2, and Rapa). Data are shown as mean ± SEM for each group (*n* = 6). Statistics table of GPLD1 protein levels in plasma. *p < 0.05 versus Con and **p < 0.01 versus Con.

To see if the increase in liver and plasma GPLD1 levels seen in long‐lived mutant mice could be induced in genetically normal mice by exposure to drugs that slow aging, we evaluated the effects of rapamycin (Harrison et al., 2009; Miller et al., 2011, 2014), canagliflozin (Miller et al., 2020), 17α‐estradiol (“17aE2”) (Harrison et al., 2014; Strong et al., 2016), and acarbose (Harrison et al., 2014), as well as the effects of caloric restriction (CR) diets. UM‐HET3 mice were placed on these drugs or diet at 4 months of age and plasma and liver harvested at 12 months. A representative immunoblot for liver tissue is shown in Figure 7 (panel a), and graphics (panels b,c) present means and SEM values for liver and plasma, taken from a set of parallel experiments using age‐matched, untreated UM‐HET3 mice as controls. Two‐way ANOVA (Sex, Drug or Diet, and Interaction) showed significant interaction terms only for Cana and 17aE2 and only in the liver. It is noteworthy that Cana and 17aE2 are the only drugs in this set that provide lifespan benefit in males only. Data for males and females were thus pooled and analyzed together for each treatment for plasma, and for Rapa, CR, and Aca for liver. In each case, the anti‐aging intervention led to a significant increase in GPLD1. For Cana and 17aE2 in the liver, *t*‐test *p*‐values were significant for males only, at *p* < 0.01 for Cana and *p* < 0.001 for 17aE2. For plasma, each of the drugs, and the CR diet, led to a significant increase in GPLD1 that was not significantly different between the sexes.

## 3 DISCUSSION

The central finding of this report is that GPLD1 levels are elevated by genes, a diet, and four drugs that extend mouse lifespan. We have also found evidence for elevation of GPLD1 in two other kinds of long‐lived mutant mice, i.e., Ames dwarf (Li et al., 2022), and PAPPA‐KO mice (Li, X., Hager, M., McPherson, M., Lee, M., Hagalwadi, R., Skinner, M., Lombard, D., Miller, R. A., unpublished data). Thus, high GPLD1 seems to be characteristic of four varieties of long‐lived mutants, four varieties of drug‐treated mice, and calorically restricted mice. We speculate that elevation of GPLD1 may be indicative of, or contribute to, processes that preserve health and postpone death as a consequence of a wide range of anti‐aging maneuvers. Our new data also show that GPLD1 production by liver reflects CIT, shown by the lack of mRNA change, the effects of a drug that promotes CIT, and the effect of a transgene that promotes CIT of selected mRNAs. We therefore propose that GPLD1 may be an important aspect of the syndrome by which diet, drugs, and mutant genes lead to extension of healthy lifespan in mice.

Previous studies have shown that Ames dwarf and GH receptor/GH binding protein knockout (GHRKO) mice, each of which is longer lived than controls, undergo age‐induced cognitive aging more slowly than their normal littermates (Kinney, Coschigano et al., 2001; Kinney, Meliska et al., 2001). Aged Ames dwarf mice have higher levels of neurogenesis in the dentate gyrus of the hippocampus (Sun, Al‐Regaiey et al., 2005), and hippocampal IGF1 protein and mRNA levels are higher in Ames mice than in controls, in contrast to the dramatically lower levels of IGF1 in Ames dwarf plasma. It is plausible, though not proven, that improved neurogenesis in Ames brain contributes to their superior cognitive performance in old age and that elevated local IGF1 production may contribute to their increased neurogenesis.

Here, we have reported higher levels of GPLD1 in liver of two varieties of long‐lived mice. Snell dwarf (DW), which are similar in endocrine profile to the Ames dwarf, and growth hormone receptor knock‐out (GKO) mice. GPLD1 is abundant in serum and leads to the release of glycosylphosphatidylinositol‐anchored proteins (GPI‐APs). There are additional GPI‐cleaving enzymes including NOTUM, glycerophosphodiesterase 2, and angiotensin‐converting enzyme prostatin, CEA, and μPAR (Hummler et al., 2013; Wilhelm et al., 1999; Yamamoto et al., 2005), which remove protein fragments from plasma membranes by hydrolysis of inositol phosphate linkages (GPI‐anchor). In parallel to the liver results, we further showed that there is a significant increase in plasma GPLD1 levels in these mice compared with their respective littermate controls. Thus, global disruption of GH signals not only increases the protein levels of GPLD1 in the liver but also increases the protein levels of GPLD1 in plasma.

Elevation of protein levels of GPLD1 in the liver was not accompanied by corresponding changes in GPLD1 mRNA in either of these long‐lived mice, suggesting that levels of GPLD1 protein may be modulated by a posttranscriptional mechanism. We have reported that GKO and Snell dwarf mice have elevated levels of many proteins that can be translated by a cap‐independent mechanism (“CIT” for cap‐independent translation) without changes in corresponding mRNA levels (Ozkurede et al., 2019). CIT‐dependent proteins are also elevated in mice exposed to drugs, including rapamycin, acarbose, and 17α‐estradiol, that increase lifespan of normal mice, suggesting that elevated CIT may represent a shared pathway for lifespan extension in mice, whether induced genetically or pharmacologically (Shen et al., 2021). This suggested that elevation of GPLD1 protein in Snell and GKO mice might also reflect preferential mRNA translation *via* CIT. To test this idea, skin‐derived fibroblasts were treated with 4EGI‐1, which diminishes normal cap‐dependent translation but increases translation of the subset of mRNAs eligible for CIT. 4EGI‐1 led to higher protein levels of GPLD1 in normal fibroblasts, but it had no effect on GPLD1 mRNA levels. Similarly, GPLD1 protein increased in the liver of normal UM‐HET3 mice that had been given oral 4EGI‐1 for 7 days. These data suggest that elevated levels of GPLD1 in the liver of slow‐aging mice may be the result of increased cap‐independent translation of GPLD1 mRNA.

To test this idea further, we measured GPLD1 levels in the liver of transgenic mice that overexpress YTHDF1. YTHDF1 promotes translation by the CIT route, by recognition of N^6^‐methyladenosine residues in the 5‐prime untranslated regions of CIT‐eligible mRNAs. We confirmed our expectation that many CIT proteins would be elevated in the liver of these YTHDF1‐transgenic mice (Figure 6b). Over‐expression of YTHDF1 also led to a two‐fold increase in liver GPLD1, showing that elevation of CIT is sufficient to increase GPLD1 in mice as well as in 4EGI1‐treated fibroblast cultures.

Horowitz and Fan have reported that Gpld1 increases in the plasma of mice after exercise and that the levels of Gpld1 correlate closely with increasing adult neurogenesis and abundance of BDNF in the aged hippocampus (Horowitz et al., 2020). We therefore speculated that increased GPLD1 in Snell and GKO mice might lead to changes in the brain that reflect GPLD1 action, and we evaluated levels of BDNF and DCX in the hippocampus. Each of these two proteins was elevated in DW and GKO mice. This seems likely to reflect increases in plasma GPLD1 because there was no change in GPLD1 protein levels in the hippocampus of slow‐aging mice. Since DCX is a marker for neurogenesis and BDNF promotes neuronal growth and survival, we speculate that the increases in these two proteins in the hippocampus may contribute to improved neurogenesis and retained cognitive function in aged GKO and DW mice.

We have shown previously that GKO mice have beneficial changes in white and brown adipocytes and in fat‐associated macrophage subpopulations (Li et al., 2020). These changes were not seen in mice with GHR disruption in fat (“FKO”) or in liver (“LKO”), suggesting that they did not reflect either direct GH action in white adipose tissue nor GH‐dependent IGF1 secretion by the liver. These changes in adipose tissue were, however, replicated in mice with muscle‐specific disruption of GHR (“MKO” mice), suggesting that the changes in adipocytes and in fat‐associated macrophages reflected GH‐dependent myokine production. We showed further that muscles of MKO and GKO mice had elevated levels of FNDC5, the precursor of the exercise‐induced myokine irisin (Li et al., 2020), and that both of these mice had elevated levels of irisin in plasma, suggesting that the changes in white and brown adipose tissues were modulated indirectly by GH‐dependent alterations in the skeletal muscle. In the current project, we used these three kinds of tissue‐specific GHR‐deficient mice to see if the increase in liver and plasma GPLD1 were dependent on GH action in muscle, liver, or adipose tissue and found no effects, suggesting that the increase in GPLD1 did not reflect GH action in any of these tissues. We also measured GPLD1 in the plasma of these mice, and found no alteration in any of these tissue‐specific GHR knockout mice. Last, we measured BDNF and doublecortin in the hippocampus of the three tissue‐specific GHR knockout mice by Western blotting and found no difference compared to WT controls. These results are consistent with the idea that alterations in BDNF and DCX in Snell dwarf and GKO mice require increased plasma GLPD1. Lifespan is unaltered in LKO mice (List et al., 2014) and slightly lower in FKO mice, and lifespan effects on MKO mice were small and were seen in only one sex and only in one of two independent experiments (List et al., 2015). We do not know what tissue or tissues respond to GH deficits in Snell DW and GKO mice to modulate liver production of GPLD1, but the GPLD1 response, and the changes in BDNF and DCX, seem to be present only in lines of mice with lifespan extension.

GPLD1 has not been linked previously to aging or cognition. Expression of GPLD1 mRNA does not change in the liver, muscle, and hippocampus with age in C57BL/6 between 6 and 18 months of age, and there were no age‐related changes in circulating levels of Gpld1 in plasma (Horowitz et al., 2020). The cited paper did not include any information on whether levels of GPLD1 protein change with age. Horowitz and Fan found that GPLD1 protein levels increased about 1.5‐fold in plasma of aged (18 months) and mature (7 months) exercised mice relative to those in plasma from sedentary age‐matched mice. Furthermore, Horowitz and Fan also found a 1.5‐fold increase in GPLD1 protein levels from active elderly human relative to sedentary counterparts. Horowitz and Fan used an in vivo transfection approach that led to a six‐fold increase in Gpld1 mRNA levels in the liver and to increased Gpld1 plasma concentrations and showed that this led to a 40% increase in hippocampal BDNF levels. This elevation of plasma GPLD1 also led to an increase in the number of newly born neurons containing doublecortin (Dcx) and an increase in the number of mature neurons expressing both BrdU and NeuN in the dentate gyrus region of the hippocampus. This group also noted the inability of plasma GPLD1 to enter the brain and that the links by which plasma GPLD1 alters brain cell pathophysiology are not understood, a topic that deserves further investigation.

GPLD1 in plasma is also elevated by Cana, 17aE2, Rapa, and Aca, as well as by CR. These changes are seen in mice tested at 12 months of age and thus seem unlikely to reflect retardation of hypothetical age‐dependent changes in GPLD1 levels. Interestingly, the effects of these drugs and dietary intervention of GPLD1 in the liver are seen in both sexes after exposure to CR, Rapa, and Aca, all of which increase lifespan in males and females, but are seen only in the males as a consequence of Cana and 17aE2 exposure. This sex‐specific pattern thus mimics the lifespan results, because Cana and 17aE2 affect lifespan only in males. Plasma GPLD1 is increased after each of these five anti‐aging interventions without a significant sexual dimorphism, and further work would be needed to learn if this disparity with the liver results reflected low statistical power to detect interaction effects or whether other tissues, besides liver, might contribute to plasma GPLD1 levels in a sex‐nonspecific way. It will be of interest to evaluate brain tissue and fat tissue of these drug‐treated mice to see to what extent changes in these tissues parallel the effects seen in Snell, GKO, and Ames dwarf mice. The mechanism that increases GPLD1 in all three sorts of mice – drug‐treated, CR, and GH/IGF1 mutant – is not fully delineated, but elevation of CIT is an obvious hypothesis.

It is currently unknown which of the many surface proteins that are susceptible to GPLD1‐mediated cleavage might contribute to its effects on brain status and on other aspects of functional homeostasis nor is it known whether GPLD1‐mediated modifications create effects by direct actions of the cleavage products or instead by alteration of the proteins from which the cleaved peptides were removed. The network of GPLD‐1‐mediated influences is likely to be tissue‐specific and is now a prime target for further exploration.

It seems plausible that drugs that increase plasma and/or liver GPLD1 levels within a few months may prove to produce lifespan benefits in mice on long‐term treatment, an idea we are now testing in a series of prospective studies. A relatively rapid screening test that can identify drugs that deserve high priority for lifespan protocols would be highly useful. Similarly, if GPLD1 levels do indeed reflect genetic or environmental variations that lead to enduring good health, we would predict that middle‐aged mice or people with relatively high plasma GPLD1 levels might be in better health and also have greater life expectancy than individuals with average of lower levels of GPLD1.

The approach we use in this project does not depend on data from aged mice because our conclusions rely on comparisons, in younger adults, between normal mice and those that are expected to age more slowly and live longer, whether their longevity is produced by diet effect, effect of single mutant genes, or drug treatments. Our inferences are based on alterations, in juvenile or young adult mice, that might contribute to slower aging, and the validity of these conclusions is not affected by any hypothetical effects of age on the endpoints we have measured, i.e., whether these endpoints go up, go down, or remain stable throughout the adult life course. Data on older animals would, however, be useful in testing ideas about the degree to which changes in GPLD1, BNDF, DCX, and CIT proteins more generally contribute to illnesses and functional deficits that become apparent in old age. Our recent study of Ames dwarf mice, including mice given injections of GH as juveniles (Li et al., 2022), shows that alterations in hippocampal BDNF and DCX, and in liver GPLD1 protein of this long‐lived mutant stock remain apparent in male mice as old as 15–18 months.

In summary, our work suggests that cap‐independent translation of GPLD1 mRNA in the liver leads, indirectly, to changes in brain proteins likely to lead to beneficial cognition effects and the resistance of these long‐lived mice to age‐related cognitive decline. Agents, like 4EGI1, that induce preferential translation of CIT mRNAs may be able to reproduce some of the pathways that delay aging and disease in long‐lived mutant and drug‐treated mice, potentially other targets of GPLD1 in the CNS and in non‐CNS tissues.

## 4 MATERIALS AND METHODS

### 4.1 Mice

Snell dwarf (DW) (homozygous dw/dw) animals (and dw/+ heterozygous littermate controls) were bred as the progeny of (DW/J × C3H/HeJ)‐dw/+ females and (DW/J × C3H/HeJ) F1‐dw/dw males. The male homozygous sires had been rendered fertile by a course of GH injections around the time of puberty. GH receptor knockout (GHRKO, here termed GKO) mice and littermate controls were bred from breeding stock originally generated by Dr. John Kopchick's group at Ohio University as previously described (Sun et al., 2009). The three tissue‐specific GHR^−/−^ mouse lines were produced by breeding GHR^flox/flox^ mice to one of three Cre‐transgenic mouse lines, each acquired from the Jackson Laboratory. The adipose tissue‐specific GHR^−/−^ mouse line (“FKO”) was generated by breeding GHR^flox/flox^ mice to B6.Cg‐Tg (Fabp4‐cre) 1 Rev/J mice. Liver tissue‐specific GHR^−/−^ mice (“LKO”) were generated by breeding GHR^flox/flox^ mice to B6.Cg‐Tg (Alb‐cre)21Mgn/J mice. Skeletal muscle‐specific GHR^−/−^ mice (“MKO”) were generated by breeding the conditional GHR^flox/flox^ mice to B6.FVB (129S4)‐Tg (Ckmm‐cre) 5 Khn/J mice. All three Cre‐recombinase transgenic mouse lines were previously backcrossed into the C57BL/6J strain; therefore, the resulting cre‐lox tissue‐specific mouse lines were a mix of C57BL/6J and C57BL/6N substrains. Breedings were coordinated in such a manner that all three tissue‐specific mouse lines used were C57BL/6 with an∼62.5% “J” and 37.5% “N” substrain mixture. The experimental protocols were reviewed and approved by the University Committee on the Use and Care of Animals at the University of Michigan.

### 4.2 Mice and experimental diets

Genetically heterogeneous UM‐HET3 mice were produced by a four‐way cross between CByB6F1 mothers and C3D2F1 fathers and housed as previously described (Garratt et al., 2017; Miller et al., 2014). Mice in breeding cages received Purina 5008 mouse chow, and weaned animals were fed Purina 5LG6. At 4 months of age, animals in different sibling groups were randomly allocated to control, Cana, CR, ACA, 17aE2, or Rapa treatments. All diets were prepared by TestDiet, Inc., a division of Purina Mills, which also produces drug/food mixtures for the NIA Interventions Testing Program. Animals in the control group remained on the 5LG6 diet. Mice in the Cana group received this agent at 180 mg/kg of chow (Miller et al., 2020). Rapamycin was given as encapsulated Rapa (L.C. Laboratories) at a dose of 14 milligrams per kilogram of 5LG6 diet (14 ppm) as previously described (Harrison et al., 2009; Miller et al., 2011, 2014). ACA was purchased from Spectrum Chemical Mfg. Corp. and used at a concentration of 1000 mg of ACA/kg of diet (1000 ppm) as previously described (Harrison et al., 2019). 17aE2 was purchased from Steraloids Inc. and used at a dose of 14.4 mg per kg of 5LG6 diet (14.4 ppm) as previously described (Harrison et al., 2014) (Harrison et al., 2019). For CR feeding: starting at 4 months of age, CR mice were given 60% of diet consumed by ad libitum control mice, as previously described (Miller et al., 2014). All these methods followed those guidelines recommended by the NIA Interventions Testing Program. All mice were treated with different diets until euthanasia at 12 months of age. In a separate experiment, young UM‐HET3 mice were given 4EGI‐1 (Millipore Sigma 324517) by daily gavage as described previously (Ozkurede et al., 2019) for 7 days prior to euthanasia.

### 4.3 Generation of YTHDF1 transgenic mice

YTHDF1 Transgenic (Tg) mice were produced in a B6SJLF1/J background by the University of Michigan transgenic core facilities. The DNA construct used for microinjection was constructed using the mouse YTHDF1‐tagMyc‐DDK (Origen plasmid MR208856) inserted into the mammalian expression vector containing the native chicken β‐actin promoter (pCAGEN, Addgene plasmid #11160) as previously described (Matsuda & Cepko, 2004) Random insertion of the linear construct into genomic DNA was confirmed by PCR as described in Figure S5d. Expression of the transgene in different tissues was confirmed by qRT‐PCR and by Western blots against MycDDK‐tag and YTHDF1 antibodies as shown in Figure 6a. F1 descendants from five founders were obtained and analyzed for the presence of the transgene. One of the Tg founders, exhibiting the highest YTHDF1 mRNA expression level, was selected for progeny generation. All the mice were used in accordance with recommendations from the Guide for the Care and Use of Laboratory Animals prepared by the Institute of Laboratory Animal Resources, National Research Council (Department of Health and Human Services Publication NIH 86‐23, 1985) and by guidelines established by the Institutional Animal Care and Use Committee at University of Michigan (Ann Arbor, MI, USA). Mice were maintained under standardized conditions with a 12‐h light/12‐h dark cycle and were provided food (standard laboratory diet) and water ad libitum.

### 4.4 Cell culture

Fibroblasts were isolated from tail tips of UM‐HET3 mice and cultured with DMEM supplemented with 10% FBS (Corning, 35011CV) and 1% antibiotics (Gibco, 15240062). Cells were kept at 37°C incubator with 10% CO_2_. Cells were treated with 4EGI‐1 at 50 μM for 24 h. 10 μM DMSO (Sigma Aldrich, D2650) was used as control.

### 4.5 RNA isolation and cDNA synthesis

Liver samples were taken from adult (4–6 months old) mice of both sexes. Samples were homogenized utilizing the Bullet Blender from Next Advance (Averill Park, NY, USA). Total RNA was isolated from mouse livers using CarbonPrep Phenol/Trizol kit (Life Magnetics, Inc.) according to the manufacturer's instruction. The RNA was cleaned using the QiagenRNeasy mini RNA cleanup protocol (Qiagen). The concentration of total RNA was performed by measuring the absorbance of RNA sample solutions at 260 nm by using a Nanodrop ND‐100. Total RNA (1.0 μg) was reverse‐transcribed using iScript cDNA reverse transcription kits (1708891; Bio‐Rad) according to the manufacturer's instructions.

### 4.6 Quantitative real‐time PCR

qPCR was performed using the Fast Start Universal SYBR Green Master Mix (Applied Biosystems). Reactions were performed using an Applied Biosystems 7500 Real‐Time RT‐PCR System. RT‐PCR was performed using quantitative PCR systems (Applied Biosystems® 7500 Real‐Time PCR Systems; Thermo Fisher Scientific) with corresponding primers (Table S1, Invitrogen). Glyceraldehyde‐3‐phosphate dehydrogenase (GAPDH) was simultaneously assayed as a loading control. The cycle time (CT) was normalized to GAPDH in the same sample. The expression levels of mRNA were reported as fold changes vs. sham control. Reactions were performed using an Applied Biosystems 7500 Real‐Time RT‐PCR System. Data were analyzed using a ΔΔ*C*_T_ approach. Table S1 lists the primers used.

### 4.7 Western blot analyses

Proteins from the liver and hippocampus were extracted after homogenization in Immunoprecipitation Assay Buffer (RIPA Buffer, Fisher Scientific) supplemented with Complete Protease Inhibitor Cocktail (Roche Inc.). Protein content was measured using a BCA assay (Fisher Scientific). The protein extracts were separated by SDS/PAGE on a 4%–15% running gel, transferred to polyvinylidene difluoride membranes, and electro‐transferred to an Immobilon‐P Transfer Membrane (Millipore) for immune blot analyses. Membranes were blocked in Tris‐buffered saline containing 0.05% Tween20 (TBS‐T) and 5% Bovine Serum Albumin (BSA) for 1 h. After blocking, membranes were probed overnight with primary antibodies in TBS‐T supplemented with 5% BSA with shaking at 4°C, followed by three 10‐min washes with TBS‐T, incubation with secondary antibody for 1 h, and three 10‐min washes with TBS‐T. Membranes were then evaluated using an ECL Chemiluminescent Substrate (Fisher Scientific). The following antibodies were used: anti‐GPLD1 (Abcam, catalog no. 210753, 1:1000), anti‐BDNF (Abcam, catalog no. 108319, 1:1000), anti‐Doublecortin (Abcam, catalog no. 18723, 1:1000), anti‐β‐actin (Santa Cruz Biotechnology, 1:1000), HRP‐conjugated anti‐mouse (GE Healthcare UK Limited, 1:2000), and anti‐rabbit (GE Healthcare UK Limited, 1:5000). Quantification was performed using ImageJ software. Table S2 lists the antibodies used.

### 4.8 Plasma GPLD1 measurement

Blood was collected from 24‐week‐old wild type littermate control mice (DWWT or GKOWT) and mutant mice (DW, GKO, LKO, FKO and MKO) into commercially available anticoagulant‐treated tubes, e.g., EDTA‐treated (lavender tops). Cells were removed from plasma by centrifugation for 10 min at 1000–2000 *g* using a refrigerated centrifuge. The resulting supernatant is designated plasma. Plasma GPLD1 concentration was determined by using a commercially available enzyme‐linked immunosorbent assay (ELISA) kit (MyBioSource, Inc.) according to the manufacturer's instructions.

### 4.9 Statistical analysis

The data shown in each figure represent results of a minimum of three independent experiments. All data are presented as mean ± SEM. Each endpoint was evaluated first by two‐factor ANOVA, using Sex, Treatment, and Interaction terms. When the interaction test was not significant, the values were combined across sex and effects of treatment evaluated by unpaired two‐tailed Student's *t*‐test. When the interaction test was significant, data were separated by sex and effects of the treatment assessed by *t*‐test separately for each sex independently. *p* < 0.05 was regarded as significant.

## AUTHOR CONTRIBUTIONS

X.L. designed and performed the experiments and wrote the manuscript. X.S. performed the cell culture experiments and edited the manuscript. M.M. and M.H. helped with experiments and edited the manuscript. G.G. created the YTHDF1 transgenic mice and participated in the studies of these transgenic animals. R.A.M. designed the experiments and edited the manuscript.

## CONFLICT OF INTEREST

The authors have no relevant conflicts of interest to declare.

## Supporting information


Figure S1

Figure S2

Figure S3

Figure S4

Figure S5

Figure S6

Table S1

Supinfo

## Data Availability

The data that support the findings of this study are available from the corresponding author on request.
