# Corrigendum to: Cap‐independent translation of GPLD1 enhances markers of brain health in long‐lived mutant and drug‐treated mice

**DOI:** 10.1111/acel.13901

**Published:** 2023-06-12

Li, X., Shi, X., McPherson, M., Hager, M., Garcia, G. G., & Miller, R. A. (2022). Cap‐independent translation of GPLD1 enhances markers of brain health in long‐lived mutant mice. *Aging Cell*, 2022, 21, e13685. https://doi.org/10.1111/acel.13685

In the original published version of the above article, the authors’ would like to expand the acknowledgement section. The corrected Acknowledgments are provided below:

