# Supplementary figures and images for: Cap‐independent translation of GPLD1 enhances markers of brain health in long‐lived mutant and drug‐treated mice
Source: Aging Cell. 2022 Aug 5;21(9):e13685. doi: 10.1111/acel.13685 (PMC9470888; doi:10.1111/acel.13685)

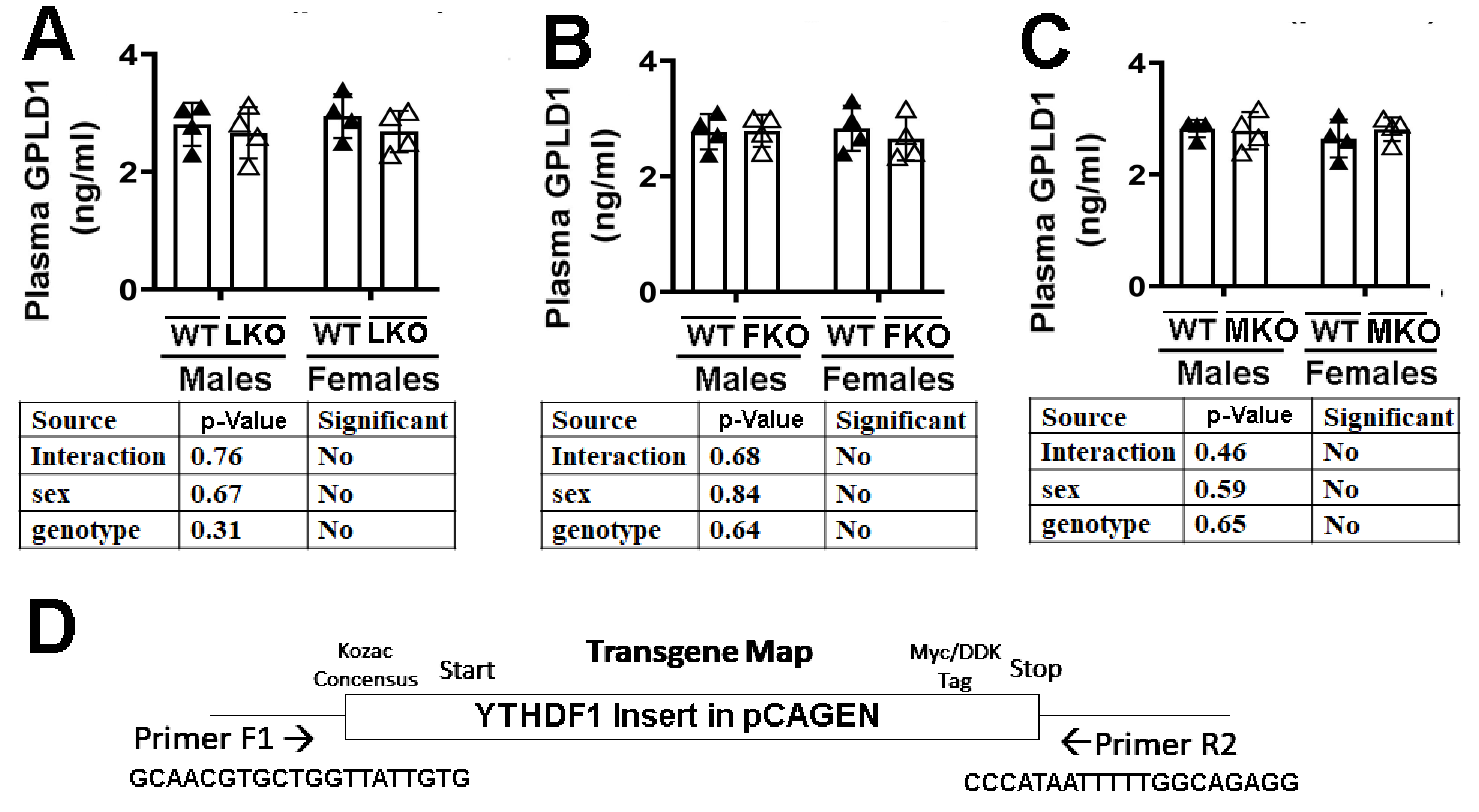

Supplement: Supplementary file 1 — Figure S1 [file ACEL-21-e13685-s005.tif]

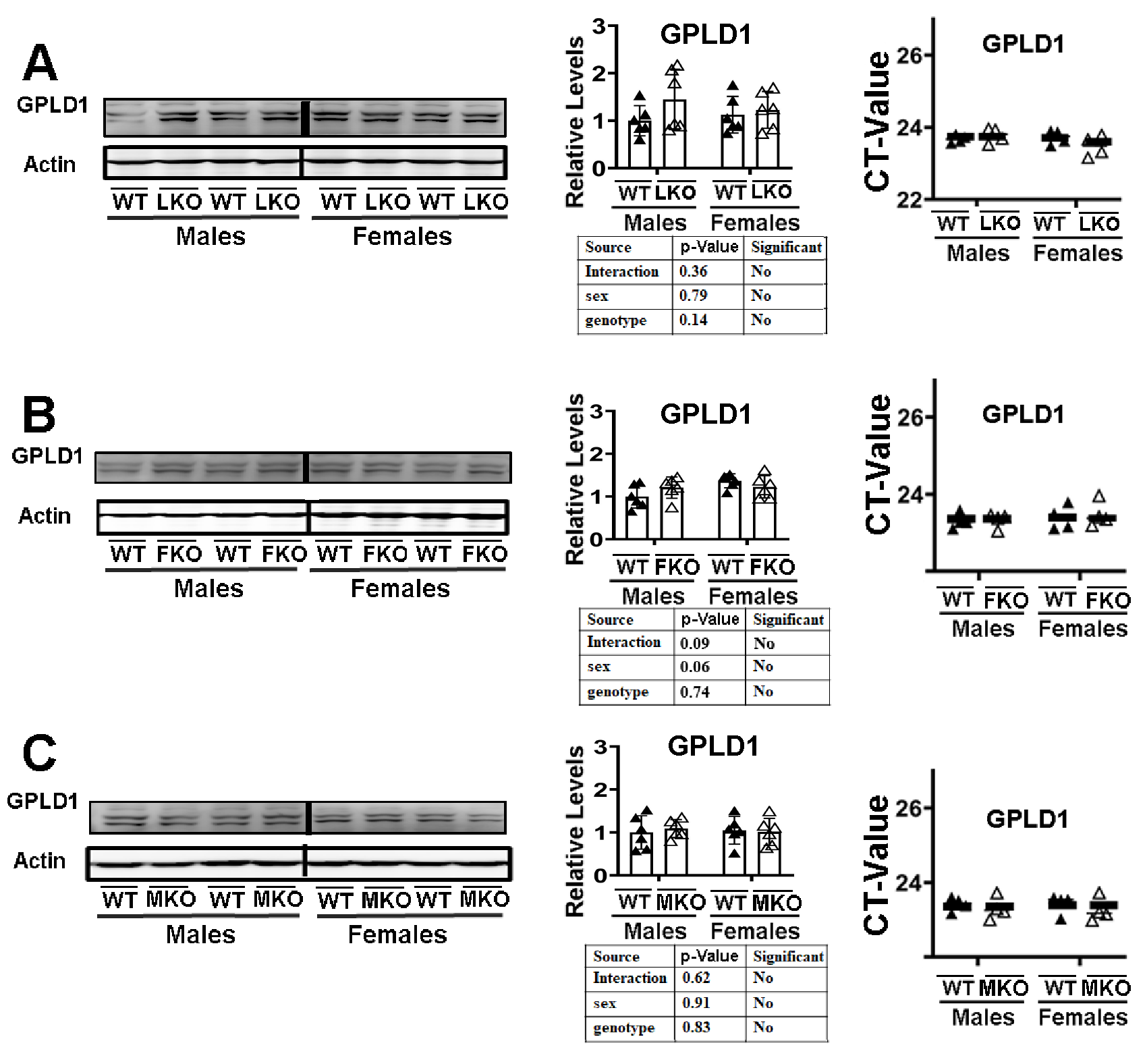

Supplement: Supplementary file 2 — Figure S2 [file ACEL-21-e13685-s001.tif]

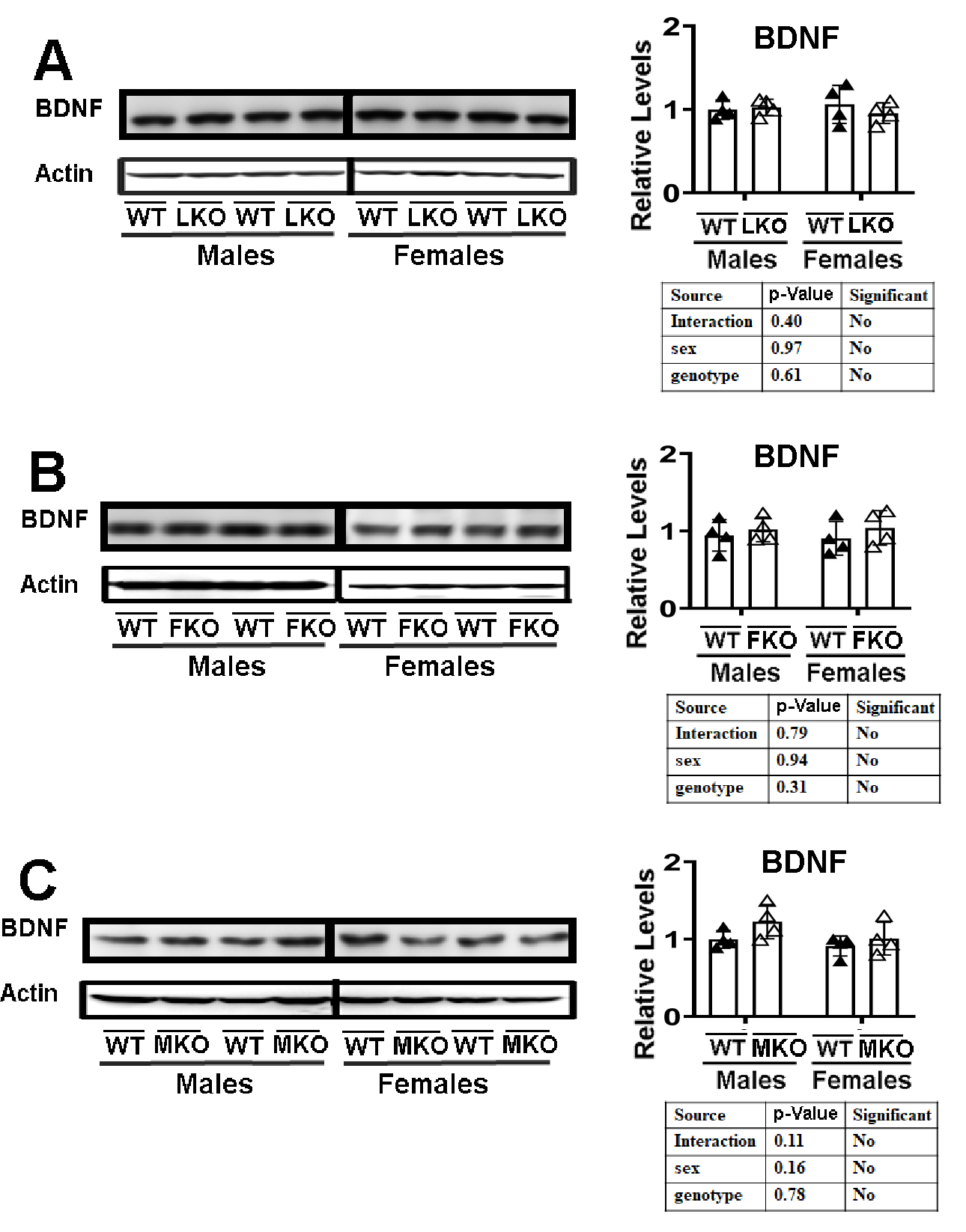

Supplement: Supplementary file 3 — Figure S3 [file ACEL-21-e13685-s008.tif]

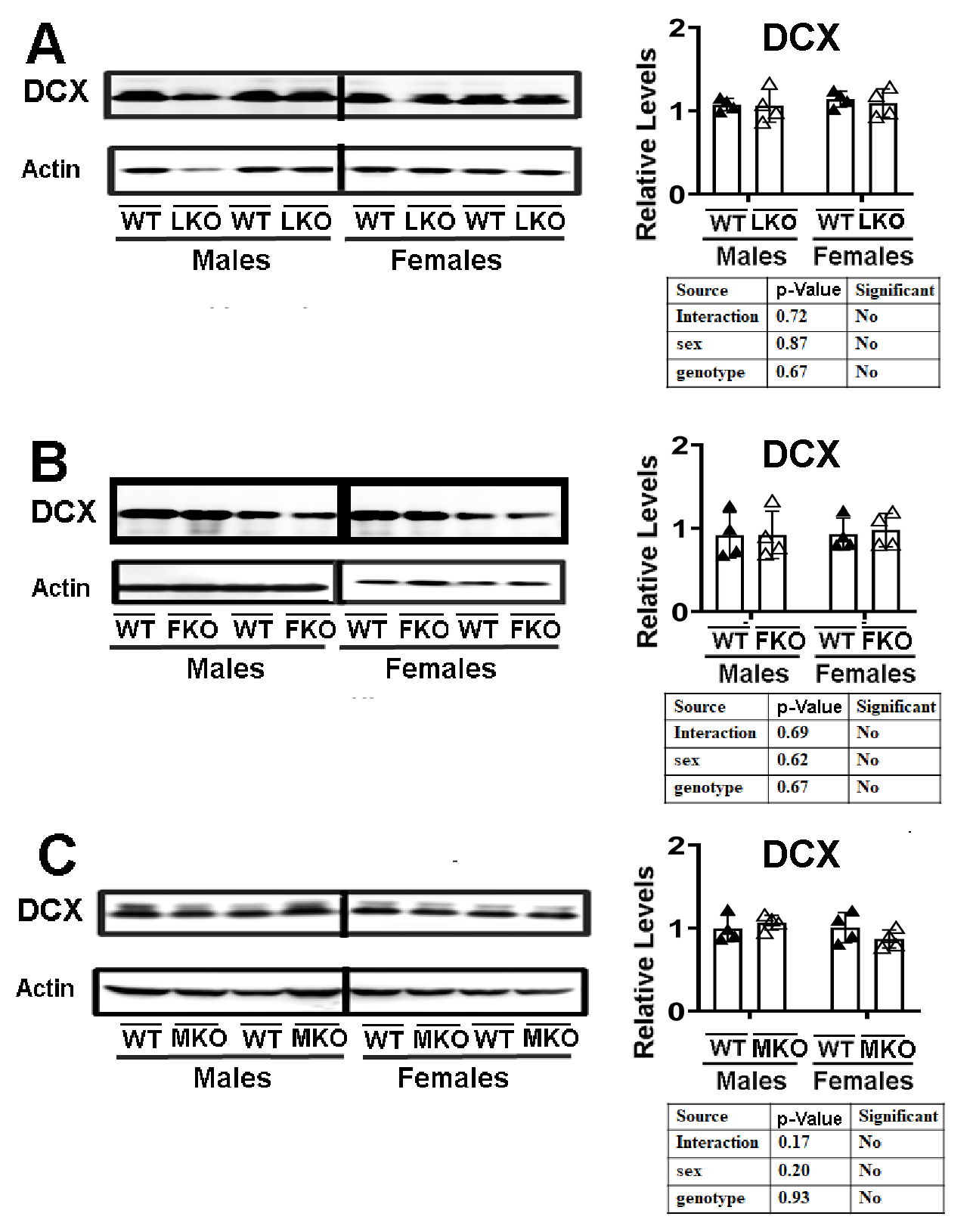

Supplement: Supplementary file 4 — Figure S4 [file ACEL-21-e13685-s002.tif]

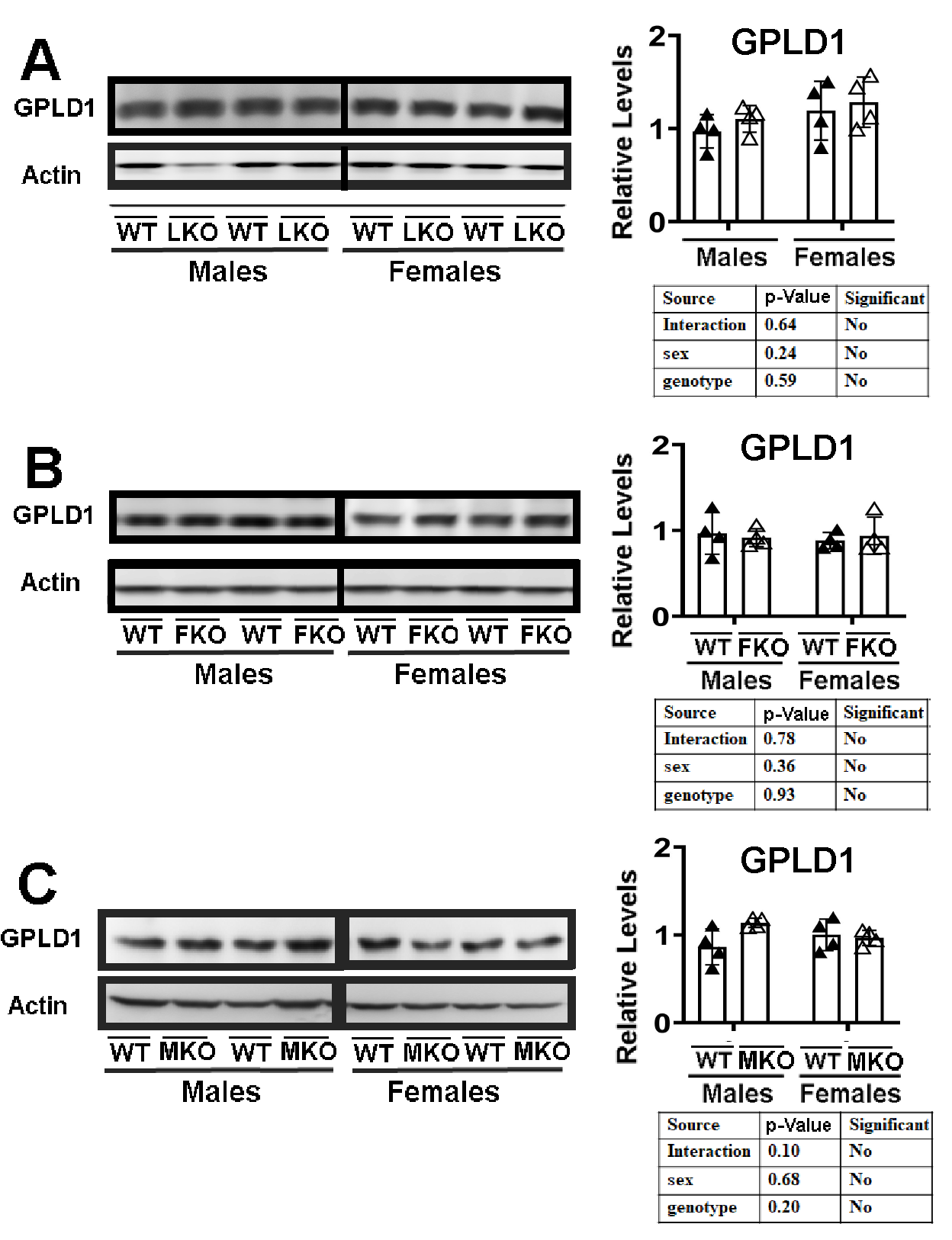

Supplement: Supplementary file 5 — Figure S5 [file ACEL-21-e13685-s006.tif]

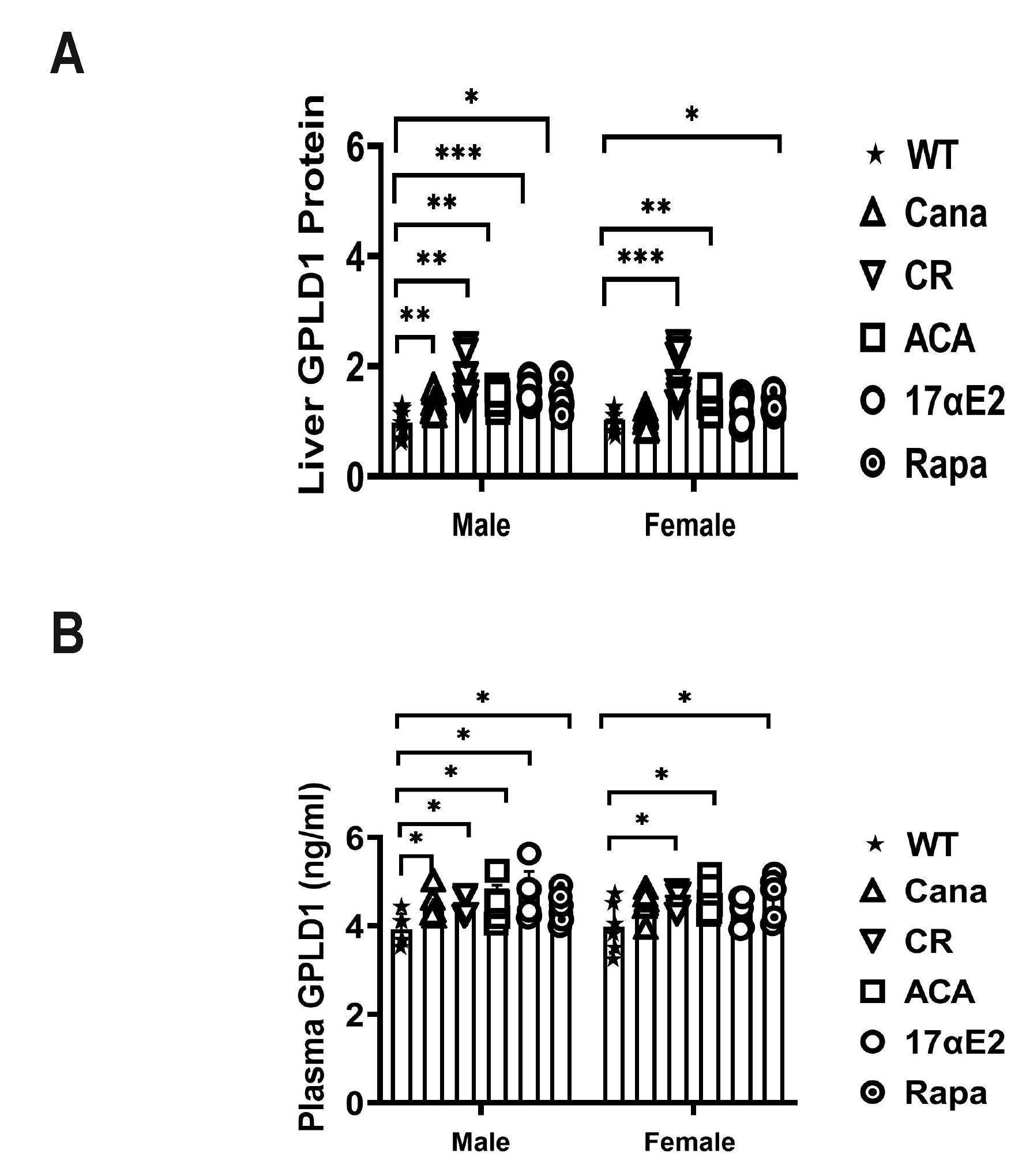

Supplement: Supplementary file 6 — Figure S6 [file ACEL-21-e13685-s004.tif]

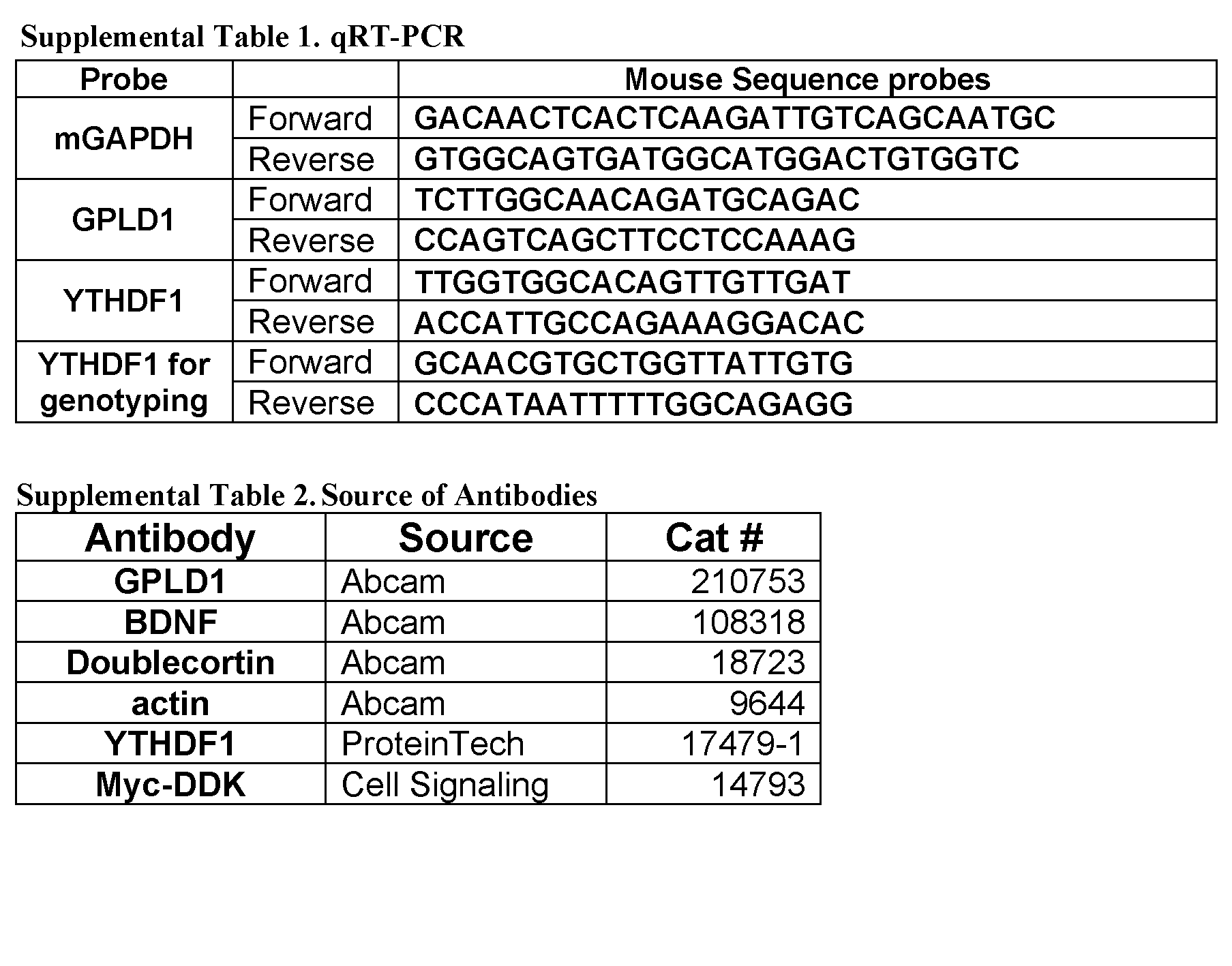

Supplement: Supplementary file 7 — Table S1 [file ACEL-21-e13685-s007.tif]
