# Supplementary material for: Cap‐independent translation of GPLD1 enhances markers of brain health in long‐lived mutant and drug‐treated mice
Source: Aging Cell. 2022 Aug 5;21(9):e13685. doi: 10.1111/acel.13685 (PMC9470888; doi:10.1111/acel.13685)
Supplement: Supplementary file 8 — Supinfo [file ACEL-21-e13685-s003.docx]

**Supplementary Figure Legends**

**Supplemental Figure 1:** **Plasma GPLD1 levels of WT (LKOWT, FKOWT and MKOWT) and mutant mice (LKO, FKO and MKO). A. B.** **C.** GPLD1 content was measured by ELISA assay on plasma samples of 24-week-old wild type littermate control mice (LKOWT, FKOWT and MKOWT) and mutant mice (LKO, FKO and MKO). Data are shown as mean ± SEM for each group (n = 4). **D.** Transgene map of YTHDF1 transgenic mice.

**Supplemental Figure 2: Effects of tissue-specific deletion of Growth Hormone Receptor (GHR) on the expression of GPLD1 in Liver Tissue.**

**A.** Total RNA and protein were isolated from liver of 24-week-old wild type littermate control mice (LKOWT) and LKO (liver deletion of GHR). **B.** Total RNA and protein were isolated from liver of 24-week-old wild type littermate control mice (FKOWT) and FKO (fat deletion of GHR). **C.** Total RNA and protein were isolated from liver of 24-week-old wild type littermate control mice (MKOWT) and MKO (muscle deletion of GHR). mRNA levels of *GPLD1* were measured by qRT-PCR. Data (mean ± SEM; n = 4) were normalized by the amount of *GAPDH* mRNA and expressed relative to the corresponding male WT value. Protein levels of liver GPLD1 were evaluated by western blotting. Representative gel images are shown. Relative protein expression was normalized to β-actin levels. Values are mean ± SEM (n = 6).

**Supplemental Figure 3:** **Effects of tissue-specific deletion of Growth Hormone Receptor (GHR) on the expression of BDNF in hippocampus.**

**A.** Cell lysate was prepared from hippocampus of 24-week-old wild type littermate control mice (LKOWT) and LKO (liver deletion of GHR). **B.** Cell lysate was prepared from hippocampus of 24-week-old wild type littermate control mice (FKOWT) and FKO (fat deletion of GHR). **C.** Cell lysate was prepared from hippocampus of 24-week-old wild type littermate control mice (MKOWT) and MKO (muscle deletion of GHR) mice. Protein levels of BDNF were then measured by western blotting. Representative gel images are shown. Relative protein expression was normalized to β-actin levels. Values are mean ± SEM (n = 4).

**Supplemental Figure 4:** **Effects of tissue-specific deletion of Growth Hormone Receptor (GHR) on the expression of DCX (doublecortin) in hippocampus.**

**A.** Cell lysate was prepared from hippocampus of 24-week-old wild type littermate control mice (LKOWT) and LKO (liver deletion of GHR). **B.** Cell lysate was prepared from hippocampus of 24-week-old wild type littermate control mice (FKOWT) and FKO (fat deletion of GHR). **C.** Cell lysate was prepared from hippocampus of 24-week-old wild type littermate control mice (MKOWT) and MKO (muscle deletion of GHR) mice. Protein levels of DCX were then measured by western blotting. Representative gel images are shown. Relative protein expression was normalized to β-actin levels. Values are mean ± SEM (n = 4).

**Supplemental Figure 5: Effects of tissue-specific deletion of Growth Hormone Receptor (GHR) on the expression of GPLD1 in hippocampus.**

**A.** Cell lysate was prepared from hippocampus of 24-week-old wild type littermate control mice (LKOWT) and LKO (liver deletion of GHR). **B.** Cell lysate was prepared from hippocampus of 24-week-old wild type littermate control mice (FKOWT) and FKO (fat deletion of GHR). **C.** Cell lysate was prepared from hippocampus of 24-week-old wild type littermate control mice (MKOWT) and MKO (muscle deletion of GHR) mice. Protein levels of GPLD1 were then measured by western blotting. Representative gel images are shown. Relative protein expression was normalized to β-actin levels. Values are mean ± SEM (n = 4).

**Supplemental Figure 6: Effects of diets treatment (Cana, CR, ACA, 17aE2 and** **Rapa) on the expression of GPLD1 in Liver and Plasma.**

**A.** Total protein were isolated from liver of 48-week-old wild type littermate control mice (Con) and diets treated mice (Cana, CR, ACA, 17aE2 and Rapa). Relative GPLD1 protein expression was normalized to β-actin levels. Values are mean ± SEM (n = 7). **p* < 0.05 versus Con. ** *p* < 0.01 versus Con and ****p* < 0.001 versus Con. **B.** GPLD1 content was measured by ELISA assay on plasma samples of 48-week-old wild type littermate control mice (Con) and diets treated mice (Cana, CR, ACA, 17aE2 and Rapa). Data are shown as mean ± SEM for each group (n = 6). Statistics table of GPLD1 protein levels in plasma. **p* < 0.05 versus Con.
